# Indicators from training healthcare providers on WHO‐recommended best practices in prenatal care in Brazil: A before‐and‐after study

**DOI:** 10.1002/ijgo.70979

**Published:** 2026-03-24

**Authors:** Sabrina O. Savazoni, Samira M. Haddad, Adriana S. Moraes, Samar A. Rahim, Mona K. Rahim, Maria T. Toro, Vitoria S. Gomes, Pedro R. Gandra, Jose G. Cecatti

**Affiliations:** ^1^ Obstetrics and Gynecology University of Campinas Campinas Sao Paulo Brazil; ^2^ Guarujá School of Medicine UNOESTE Guarujá Sao Paulo Brazil

**Keywords:** management indicators, maternal health, perinatal health, prenatal care, process indicators

## Abstract

**Objective:**

To analyze prenatal indicators of management, process, and obstetrical outcomes, before and after training healthcare providers on the WHO recommendations for best evidence‐based practices.

**Methods:**

A quasi‐experimental before‐and‐after operational study was conducted from January to July 2022. The study occurred in seven healthcare units and one municipal hospital on the coast of Brazil. Medical charts of all pregnant women receiving prenatal care in the sample units during this period were reviewed. Hospital data collection was conducted, with medical chart review of all postpartum women who had given birth in January and June and came from the same sample units. All healthcare professionals of the sample units participated in training focused on the best prenatal practices. For data analysis, the *χ* ^2^‐test, Fisher exact test and Student *t*‐test were used, with a significance level of *P* values less than 0.05.

**Results:**

In all, 568 medical charts were studied, 278 in the “before” and 290 in the “after” training period. After training, a significant improvement occurred with records of increased screening for: anemia (from 74% to 92%), HIV (from 95% to 98%), diabetes (from 75% to 93%), asymptomatic bacteriuria (from 74% to 88%), and ultrasound scan before 24 weeks of pregnancy (from 74% to 85%), although the management record of pregnant women with anemia was worse (from 85% to 45%).

**Conclusion:**

Training was efficient in terms of process indicators. After staff training, there was a significant increase in documented screening for main maternal clinical conditions, including anemia, diabetes, and asymptomatic bacteriuria, although clinical management did not consistently improve.

## 1 INTRODUCTION

Maternal and infant morbidity and mortality have been a globally prioritized theme since the 1980s. The Sustainable Development Goals of the United Nations aim for a global maternal mortality ratio (MMR) lower than 70 per 100 000 live births by 2030, and the Brazilian goal is 30 per 100 000 live births.^1^, ^2^, ^3^ In 2020, the global MMR was 223 per 100 000 live births, with 92.3% of deaths occurring in low‐ and middle‐income countries, highlighting regional inequalities, such as sub‐Saharan Africa (545 per 100 000 live births) and Australia (4 per 100 000 live births).^1^, ^4^

The COVID‐19 pandemic aggravated these inequalities, with significant increases in MMR, such as in the region called Baixada Santista on the coast of Sao Paulo state in Brazil, ranging from 49.03 to 146.98 per 100 000 live births between 2019 and 2022.^5^, ^6^ Despite advances, maternal and infant death rates continue above the expected, particularly in vulnerable populations. Poor quality of care and social determinants may be the cause of these inequalities.^4^, ^7^, ^8^

Prenatal care consists of clinical and educational procedures that aim to accompany the pregnancy, promote the health of the pregnant woman and baby, identify risk factors, treat complications and ensure adequate referral according to individual needs.^9^, ^10^ In addition to the prevention and early diagnosis of conditions that contribute to maternal complications, prenatal care addresses issues such as unwanted pregnancies, unsafe nutrition, sexually transmitted infections, and inadequate family planning.^11^

One of the leading elements for the effectiveness of prenatal care is the adherence of the woman to care, although this depends not only on the woman herself but also on the quality, accessibility, and acceptability of the care. There is a correlation between adherence to and the quality of care provided by the service and healthcare professionals.^12^ This is to reduce hypertension, hemorrhage, infections, and unsafe abortions, which are the main causes of maternal morbidity and death.^13^ Prenatal consultation should offer a space for active listening and support, strengthening the autonomy of the pregnant woman. Nevertheless, delays in access to care, failures in communication between services and adoption of treatment that is not based on evidence can hinder care and result in avoidable deaths.^7^ Prenatal care is a fundamental opportunity to identify risks, prevent unfavorable outcomes, and contribute to the reduction in maternal and perinatal morbidity and mortality.^13^, ^14^

Even in low‐developed and low‐income settings, professional training in prenatal care has positive results. This includes the engagement of pregnant women and the provision of evidence‐based care, despite barriers such as time constraints and limitations in training.^15^, ^16^

In 2016, WHO published the guideline *WHO recommendations on prenatal care for a positive pregnancy experience*. It contains 49 evidence‐based recommendations to ensure prenatal care of good quality, considering factors such as values, equality, resources, and viability.^16^ Nevertheless, its implementation may be limited without efficient strategies and knowledge about local indicators, essential for the development of targeted actions.

Both WHO and the Brazilian Ministry of Health emphasize the surveillance of prenatal indicators as a strategy to improve care and reduce maternal and infant mortality. Furthermore, the training of healthcare staff is crucial to ensure high‐quality, integral care for pregnant women, promoting maternal and neonatal health.^4^

Primary care professionals have a crucial role in healthcare improvement. Strengthening these services is the most cost‐effective approach to improving health outcomes and reducing maternal morbidity and mortality.^11^ Strengthening primary care does not necessarily demand elevated costs or advanced technology, but it does require continuous training of healthcare staff and monitoring of health indicators to reduce disparities, achieve universal coverage and improve maternal–infant outcomes, as pointed out by WHO.^17^

In this context, the current study aims to analyze prenatal indicators of management, process, and screening practices, before and after staff training in WHO recommendations on best evidence‐based practices to be implemented in routine prenatal care in Guarujá, a coastal municipality that has the worst maternal and perinatal health indicators in the richest state of São Paulo, Brazil. It is believed that this training would improve the health indicators and screening practice, thus helping in the qualification of healthcare and possibly reducing maternal and infant morbidity and mortality in this region.

## 2 MATERIALS AND METHODS

### 2.1 Study design

This is a prospective quasi‐experimental before‐and‐after operational study that evaluated indicators of maternal and perinatal management, process and outcomes before and after the training of health providers, from January 2022 to July 2022, for the implementation of the WHO best practices in prenatal care in a municipality of the state of São Paulo, Brazil. The “before training” group was the control for the “after training” group.

### 2.2 Sample and setting

Data were collected from the medical charts of pregnant women managed in six Family Healthcare Units (USAFAs), one secondary care unit (Casa Rosa) and the Santo Amaro Hospital (HSA), in the municipality of Guarujá, SP. The selection was not probabilistic but used a convenience sampling method, encompassing units linked to the Guarujá School of Medicine (UNOESTE). The USAFAs managed low‐risk pregnant women, Casa Rosa accompanied high‐risk cases, and HSA, a philanthropic tertiary hospital, managed high‐risk pregnant women, with adult and neonatal intensive care unit support.

### 2.3 Data collection

Data from medical charts were reviewed at two time periods: January 2022 (before) and June 2022 (after training). Complementary hospital information was collected in December 2022 and January 2024. Data collection used an online form with variables to calculate process and management indicators, outcomes and prevalence of morbid conditions, ensuring anonymity and confidentiality. Results were not disclosed to the healthcare staff during the study period to minimize the effect of the observer (Hawthorne effect).

### 2.4 Professional training

The USAFA and Casa Rosa health professionals were trained on WHO recommendations for prenatal care, in partnership with the Guarujá School of Medicine and the Health Secretariat of the municipality. Twenty‐eight professionals participated (14 family physicians who are responsible for providing prenatal care, 10 nurses and 4 municipal managers), corresponding to 100% of the target population. In‐person training, conducted on the local university campus by the professors and health professionals from the Health Secretariat, had a duration of 2 h and was structured in five presentations of 15 min, followed by questions and answers.

Six medical students, under strict supervision by professors and by the Health Secretariat to guarantee the depth and appropriateness of the material, developed and adapted the training content based on state, municipal, and WHO protocols. Topics included consultation routine, prenatal tests, anemia, diabetes, urinary tract infections, hypertension, HIV, and syphilis.

### 2.5 Data analysis

For comparisons between groups, *χ* ^2^ or Fisher exact tests were used. To compare numerical variables between two time periods, a Student *t*‐test was used. Missing data were considered in the analysis, and no imputation was performed. The level of significance was set at a *P* value less than 0.05. For the analysis procedures, the Epi.Info™ (version 7.2, CDC, Atlanta, GA, USA, 2024) and SPSS Statistics (version 30, IBM, Armonk, NY, USA, 2024) packages were used.

### 2.6 Ethical aspects

The study was approved by the Ethics Committee of the institution Associação Prudentina de Educação e Cultura APEC (CAAE: 51325121.0.0000.5515, Letter of approval number 5.162.276 from December 14, 2021). For participation in the prenatal training program, health professionals eligible to participate in the study signed a written informed consent term after an explanation about the study and their participation. For women participating in the study, the informed consent term was waived because data were collected retrospectively from their medical charts.

## 3 RESULTS

**FIGURE 1:**
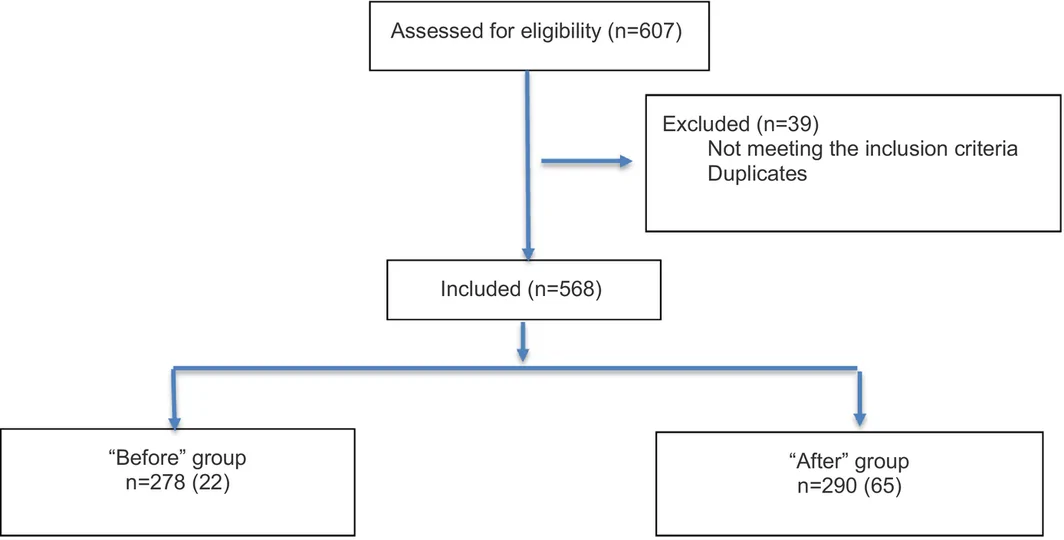
Total data collected from 607 cases. After the exclusion of 39 duplicate cases: The “before” group consisted of 278 pregnant women: 256 prenatal and 22 outcome pregnant women. In contrast, the “after” group consisted of 290 women: 225 prenatal and 65 outcome pregnant women.

Information was collected from 607 medical charts of eligible women. Of these, 39 women had their medical charts reviewed in both periods, before and after training. These duplicate cases were analyzed only in the period after training, excluding data from the period “before” training. Hence, the total number of cases included was 568 women, with 278 cases in the period before training (22 of these women with outcome data) and 290 in the period after training (65 of these with outcome data). Figure 1 shows the flow chart of inclusion.

Sociodemographic data are shown in Table 1. The groups were shown to be homogeneous, with no significant differences between them. The majority of women (414, 74%) were between 20 and 34 years old. Eleven women were between 11 and 16 years old (2%), all of whom were primipara, and only three (27%) had a partner. Seventy‐five percent of the primiparas were between 11 and 25 years of age. There was early recruitment (≤12 weeks) in 78% (446) of the total sample. There was no significant difference in obstetrical variables between periods.

**TABLE 1:** Sociodemographic and obstetrical characteristics of pregnant women before and after healthcare providers training.^a^

Characteristics	Before	After	*P*‐value
Maternal age, year			0.804
<20	33 (12.09)	30 (10.53)	
20–34	202 (73.99)	212 (74.39)	
≥35	38 (13.92)	43 (15.09)	
Marital status			0.588
With partner	173 (62.23)	179 (61.72)	
Without partner	76 (27.34)	87 (30.00)	
No record	29 (10.43)	24 (8.28)	
Schooling			0.981
No schooling	01 (0.36)	01 (0.34)	
Fundamental	34 (12.23)	36 (12.41)	
High school	125 (44.96)	127 (43.79)	
Superior	26 (9.35)	24 (8.28)	
No record	92 (33.09)	102 (35.17)	
Number of pregnancies			0.273
1	122 (43.88)	108 (37.24)	
2–3	121 (43.53)	141 (48.62)	
≥4	30 (10.79)	37 (14.14)	
No record	5 (1.79)	4 (1.37)	
Gestational Age at the first visit			0.839
≤12 weeks	222 (79.86)	224 (77.24)	
13–19 weeks	33 (11.87)	36 (12.41)	
≥20 weeks	22 (7.91)	19 (6.55)	
No record	1 (0.36)	11 (3.79)	

^a^ Data are presented as number (percentage). Total of 568 women—278 in the period before and 290 in the period after training.

Table 2 shows the prevalence of complications analyzed during the prenatal and postpartum periods. Of the total sample, about 15% of women had some comorbid condition or risk criteria. Except for HIV, all clinical conditions had a prevalence above 5%, highlighting bacteriuria (22%), hypertension (10%), and syphilis (9%). Data on perinatal outcomes were limited to a small number of cases.

**TABLE 2:** Prevalence of clinical conditions during pregnancy and perinatal outcomes before and after healthcare providers training.^a^

Clinical conditions/perinatal outcomes	Before	After	*P*‐value
Clinical conditions			
Anemia			0.674
Yes	20 (7.19)	20 (6.90)	
No	170 (61.15)	168 (57.93)	
No record	88 (31.65)	102 (35.17)	
Maternal syphilis			0.361
Yes	28 (10.07)	25 (8.62)	
No	238 (85.61)	258 (87.32)	
No record	12 (4.32)	7 (2.41)	
HIV			0.069
Yes	0	2 (0.69)	
No	265 (95.32)	282 (97.24)	
No record	13 (4.68)	6 (2.07)	
Diabetes			0.762
Yes	17 (6.12)	16 (5.52)	
No	171 (61.51)	187 (64.48)	
No record	90 (32.37)	87 (30.00)	
Hypertension			0.210
Yes	25 (8.99)	32 (11.03)	
No	245 (88.13)	255 (87.93)	
No record	8 (2.88)	3 (1.03)	
Bacteriuria			0.064
Yes	70 (25.18)	55 (18.97)	
No	117 (42.09)	138 (47.59)	
No record	91 (32.73)	97 (33.45)	
Perinatal outcomes			
Gestational age at birth			0.060
≤28 weeks	1 (3.33)	0	
29–36 weeks	0	5 (7.58)	
≥37 weeks	28 (93.33)	61 (92.42)	
No record	1 (3.33)	0	
Birth weight			**0.020**
1001–1500 g	1 (3.33)	0	
1501–2499 g	2 (6.67)	7 (10.61)	
≥2500 g	24 (80.00)	59 (89.39)	
No record	3 (10.00)	0	
Infant death			0.509
No	26 (86.66)	65 (98.48)	
Yes <2500 g	0	0	
Yes ≥2500 g	1 (3.33)	1 (1.51)	
No record	3 (10.0)	0	

^a^ Data are presented as number (percentage). Total of 568 women—278 in the period before training and 290 in the period after training. Total of 96 women in the postpartum—30 in the period before and 66 in the period after training. Values in bold are statistically significant.

Concerning obstetrical outcomes, six women had preterm deliveries (6.25%), and 10 newborn infants had low birth weight (10.41%). Among the preterm babies, maternal age ranged from 18 to 42 years (median: 26 years), five were late preterm babies (after 34 weeks of pregnancy), and three women had 11 prenatal visits. Both infant deaths occurred in 38‐week pregnancies, and newborn infants weighed between 2970 and 3130 g (data not shown in tables).

Process indicators of prenatal care are listed in Table 3. Screening rates for the diverse conditions ranged from 79.9% to 98% of the records. An increase of 18.7% in screening for anemia was highlighted, followed by a 17.2% increase for diabetes and a 13.7% increase for asymptomatic bacteriuria in the before group in comparison to the after group. The prevalence of women who initiated prenatal care up to 12 weeks of pregnancy was 78.5%. Regarding the adequacy of the number of visits (at least eight visits for a term pregnancy), 82.3% of the women had an adequate number for gestational age at the time of analysis.

**TABLE 3:** Process indicators—basic recommendations for prenatal care before and after healthcare providers training.^a^

Process indicators		Before	After	*P*‐value
Iron supplementation	Yes	194 (69.78)	219 (75.52)	0.132
	No record	84 (30.22)	71 (24.48)	
Folic acid supplementation	Yes	213 (76.62)	230 (79.31)	0.479
	No record	65 (23.38)	60 (20.69)	
Screening for anemia	Yes	205 (73.74)	268 (92.41)	**<0.001**
	No record	73 (26.26)	22 (7.59)	
Screening for syphilis	Yes	266 (95.68)	285 (98.28)	0.070
	No record	12 (4.32)	05 (1.72)	
Screening for HIV	Yes	266 (95.68)	286 (98.62)	**0.042**
	No record	12 (4.32)	4 (1.38)	
Screening for diabetes	Yes	210 (75.54)	269 (92.76)	**<0.001**
	No record	68 (24.46)	21 (7.24)	
Screening for hypertension	Yes	270 (97.12)	287 (98.97)	0.135
	No record	8 (2.88)	3 (1.03)	
Screening for bacteriuria	Sim	207 (74.46)	256 (88.28)	**<0.001**
	No record	71 (25.54)	34 (11.72)	
Ultrasound before 24 weeks of pregnancy	Yes	207 (74.46)	247 (85.17)	**0.001**
	No	10 (3.60)	12 (4.14)	
	No record	61 (21.94)	31 (10.69)	
Early onset of prenatal care (≤12 weeks)	Yes	222 (79.86)	224 (77.24)	0.966
	No	55 (19.78)	55 (18.97)	
	No record	1 (0.36)	11 (3.79)	
Adequacy of the number of prenatal visits	Yes	232 (83.45)	241 (83.10)	0.121
	No	42 (15.11)	37 (12.76)	
	No record	4 (1.44)	12 (4.14)	

^a^ Data are presented as number (percentage). Total of 568 women—278 in the period before training and 290 in the period after training. Values in bold are statistically significant.

The detection rates of clinical conditions (positivity ratio among screened women) are shown in Table 4. There was a very small variation in the detection of conditions studied after training, except for hypertension, whose detection increased slightly.

**TABLE 4:** Detection rate of clinical conditions before and after health professional training.^a^

Clinical condition	Before	After	Total
Anemia	9.75	7.46	8.45
Syphilis	10.52	8.77	9.61
Diabetes	8.09	5.94	6.88
Hypertension	9.25	11.1	10.23
Bacteriuria	33.81	21.48	26.99

^a^ Data are presented as percentages. Detection rate = number of women positive for the condition/number of screened women.

Table 5 shows the results for adequacy rates of diagnoses of complications and management of positive cases. An adequate diagnosis was considered when positive or negative cases were adequately identified. Non‐adequacy is considered false‐positive and false‐negative cases. Unexpectedly, among the complications assessed, anemia showed a lower adequacy of management after training, possibly an effect of the low number of cases, which should be taken into account.

**TABLE 5:** Management Indicators—adequacy of diagnosis and management of clinical conditions before and after healthcare providers training.^a^

Indicator	Adequacy of management	Before	After	*P*‐value
Diagnosis of anemia	Yes	188 (67.63)	184 (63.45)	0.295
	No record	90 (32.37)	106 (36.55)	
Management of anemia	Yes	17 (85.00)	09 (45.00)	**0.019**
	No record	3 (15.00)	11 (55.00)	
Diagnosis of syphilis	Yes	259 (93.17)	278 (95.86)	0.157
	No record	19 (6.83)	12 (4.14)	
Management of syphilis	Yes	21 (75.00)	20 (80.00)	0.750
	No record	7 (25.00)	5 (20.00)	
Diagnosis of HIV	Yes	265 (95.32)	284 (97.93)	0.084
	No record	13 (4.68)	06 (2.07)	
Management of HIV	Yes	00.00	02 (100)	**<0.001**
	No record			
Diagnosis of diabetes	Yes	188 (67.63)	199 (68.62)	0.799
	No record	90 (32.37)	91 (31.38)	
Management of diabetes	Yes	10 (58.82)	11 (68.75)	0.554
	No record	7 (41.18)	5 (31.25)	
Diagnosis of hypertension	Yes	260 (93.53)	274 (94.48)	0.631
	No record	18 (6.47)	16 (5.52)	
Management of hypertension	Yes	26 (104)	20 (36.36)	0.492
	No record	12 (48)	13 (40.62)	
Diagnosis of bacteriuria	Yes	178 (64.03)	184 (63.45)	0.982
	No record	100 (35.97)	106 (36.55)	
Management of bacteriuria	Yes	66 (94.28)	48 (87.27)	0.138
	No record	8 (11.42)	12 (21.81)	

^a^ Data are presented as number (percentage). Total of 568 women—278 in the period before and 290 in the period after the training. Values in bold are statistically significant.

Concerning management, adequacy took into account treatment for positive cases or lack of records for negative cases. Non‐adequate cases refer to management for positive cases that were not recorded or when management was recorded for negative cases.

There was a decrease in records of management of anemia in the period following training. There was no significant difference for the other conditions. There was an increase in the diagnosis and management of syphilis and diabetes, and the diagnosis of hypertension. However, there was a decrease in adequacy for the diagnosis and treatment of bacteriuria and the management of anemia.

## 4 DISCUSSION

The results of the present study revealed important gaps in the medical records and care provided to pregnant women in the units analyzed. Incomplete, missing, or equivocal records were a significant challenge and could be understood as a measurement bias. Non‐recorded information was treated as a lack of care and had a direct impact on data analysis. For example, information about anemia was absent in 33.4% of the medical charts, and data on asymptomatic bacteriuria and gestational diabetes were absent in 33.1% and 31.1%, respectively. This reality reflects the important fragility in the management of information in healthcare units and limitations in the integration among different levels of care.

Concerning the prevalence of anemia in pregnancy, the present study found a rate of 7.04%. This value was significantly lower than the global mean rate of 36.5% and the mean Brazilian rate of 19.1%, according to WHO estimates.^18^ This finding may be partially explained by the high supplementation rate of iron and folic acid recorded in the sample (more than 70%), in addition to potential sociodemographic differences among the women receiving care. However, a lack of records in a third of the medical charts undermines the reliability of these data and prevents a more accurate evaluation.

Maternal syphilis was another indicator of the quality of prenatal care that was analyzed in the present study. The prevalence of maternal syphilis in the sample was 9.33%. This rate was about threefold higher than the national mean of 3.4% and much higher than global estimates of 0.69%.^19^, ^20^ These data suggest significant fragility in prenatal care, especially in diagnosis and proper management. In 22.6% of the pregnant women diagnosed with syphilis, necessary care, including antibiotic therapy and counseling, was not provided or recorded, or was equivocal, indicating the avoidance of many cases of congenital syphilis. These numbers reinforce the importance of continuous training and more active surveillance of this disease, especially considering its avoidable negative impact on neonatal outcomes.

Regarding asymptomatic bacteriuria, the overall prevalence ranges from 2% to 15% of pregnant women, according to previous studies.^21^, ^22^ The present study showed a prevalence of around 22%, much above the global prevalence. However, it highlighted significant improvements in screening after training, increasing from 74.46% to 88.28%. Nevertheless, problems were identified, including inadequate antibiotic prescription for women with negative cultures, which may indicate a misdiagnosis based on less specific tests, such as the urinary sediment test (urine 1). Untreated asymptomatic bacteriuria has repercussions, such as prematurity and maternal sepsis. These effects reinforce the need for consistent interventions, including rapid access to laboratory test results and timely initiation of adequate treatment.

Gestational hypertension was identified in 10.04% of the pregnant women in the sample, a value higher than the national average of 7.5% reported by Brazilian population‐based studies.^23^ On the other hand, SUS (Unified Health System) data indicate a national prevalence of pre‐eclampsia of 2.3%, while eclampsia occurs in 0.6% of pregnancies.^24^ National reports demonstrate that there are few population‐based studies published specifically on pre‐eclampsia in Brazil.^25^ In Latin America, pre‐eclampsia affects from 2% to 8% of all pregnancies and is responsible for one‐quarter of all maternal deaths in the region.^26^ Hypertension in pregnancy is one of the main risk factors for maternal mortality, accounting for up to 25% of maternal deaths in Brazil.^27^ These numbers reflect the need for greater surveillance and proper management of hypertension in primary and secondary care, which may have a potential impact on maternal and neonatal outcomes. With actions to reduce maternal and infant morbidity and mortality rates due to pre‐eclampsia, pregnant women at risk for developing the disease should be identified, encouraged to engage in physical activity, administered acetylsalicylic acid, and supplemented with calcium.^28^ A more encompassing study to analyze the impact of screening and secondary prevention of pre‐eclampsia is necessary during pregnancy, delivery, and the postpartum period.

Concerning gestational diabetes, the prevalence in the sample was 5.81%, slightly lower than the global mean rates, which range from 2% to 38%, depending on the criteria used.^29^ In the United States, for example, the prevalence rate was 7.8% in 2020, according to criteria established by Carpenter and Coustan.^30^ Although screening for diabetes has improved from 75.54% to 92.76% after staff training, proper management of diagnosed cases still depends on improvement in flow care. This includes timely laboratory specimen collection and interpretation of test results, along with the availability of inputs for monitoring.

Prematurity, births occurring before 37 weeks of pregnancy, was recorded in 6.25% of the analyzed cases. This value aligns with the global mean rate of around 10%, according to Dagklis et al.^31^ The majority of preterm births in the sample occurred between 29 and 36^+6^ weeks, and the earliest case was recorded at 27 weeks. The latter involved a pregnant woman with well‐managed gestational diabetes, whose newborn infant survived. These data reinforce the importance of continuous monitoring during pregnancy, especially in high‐risk cases.

The present study also identified positive aspects of prenatal care, such as a high rate of early recruitment (78.52%) and initiation of prenatal care before 20 weeks of pregnancy in 90.67% of pregnant women. Early recruitment is an important indicator as it enables the healthcare providers to have a better organization and treatment plan. These data are positive in comparison to a nationwide hospital study by Domingues et al.^32^ that evidenced early recruitment in the prenatal period in 53.9% between 2011 and 2012. Nevertheless, the quality of interaction during consultations seems to be a critical factor for a positive impact on outcomes, and it is a difficult aspect to measure quantitatively.

Finally, two neonatal deaths were recorded, one in each group (“before” and “after” training). Both involved term newborn infants, and there were no significant maternal comorbid disorders. These cases highlight the need for a more integrated, continuous approach that considers not only prenatal care but also care at the time of delivery and during the postpartum period. Avoidable deaths could thus be prevented.

Short‐term training, although modest, improved process indicators. Screening for anemia increased from 73.74% to 92.41%; gestational diabetes increased from 75.54% to 92.76%; and asymptomatic bacteriuria increased from 74.46% to 88.28%. Only one indicator, management of anemia, was worse after training, demonstrating that simple interventions can improve care. Whether the training failed to cover follow up and case management sufficiently or whether implementation barriers, such as medication access and laboratory delays, were present remains hypothetical because these aspects were not covered. Nevertheless, robust changes in outcomes require training in urgent and emergency obstetrial care.

As limitations, data collection was hindered by incomplete records and hybrid systems (handwritten and digital), making it difficult to integrate information between levels of care. The local hospital faced logistical and administrative challenges, such as external filing of medical charts, which limited outcome analysis. Some of the high‐risk patients managed in the secondary unit were excluded from the study because of the lack of clear information about the primary referral units. In addition, it could be argued that the time for and type of training were not the most appropriate for this objective.

Other limitations were the low number of women with outcomes available, the non‐probabilistic and convenience‐based sampling, and the lack of a representative sample of the municipality. Although the included units corresponded to 40% of the USAFAs, missing data had an impact on the complete evaluation of the situation. However, the counterpoint here is that the reasons why and how the study was implemented—as a previous step for then introducing a new digital application from WHO for prenatal care specifically in this setting—could probably not be differently performed, considering it included all the health professionals involved in the selected health units (the same from where the applications was planned to be implemented) and no more cases could be included in these units because of the incompleteness of data of clinical records.

The lack of integration between chart systems indicates the need for simple and encompassing digital tools. A digital solution could include automated alerts for altered laboratory results and clinical guidance for healthcare providers, optimizing the management of information and quality of care.

There is a demand in the municipal healthcare system to broaden the capacity for high‐risk prenatal care. The shortage of specialists and delays in access to specialized consultation may compromise maternal and perinatal health. Partnerships with universities may offer the opportunity to redesign processes, relieving the demand for specialized consultations, sharing qualified human resources, and investing in infrastructure.

Periodic training and public policies aimed at retaining professionals are also essential to avoid a high turnover rate and ensure continuity in staff training. Regional laboratory specimen collections without the need for scheduling could reduce delays in diagnoses of conditions such as anemia and gestational diabetes.

In conclusion, the results of the present study, although limited by issues with medical records and lack of a real representative sample, demonstrate that this short training improved the documentation of screening indicators but had limited or no impact on the clinical management of maternal conditions. Increased screening for important conditions, such as anemia, diabetes, HIV, asymptomatic bacteriuria, and ultrasound scans performed before 24 weeks of pregnancy, were particularly positively affected by this intervention, although the impact on management was mostly non‐significant, and with a reduction in providing adequate treatment to cases diagnosed with anemia. These findings reinforce the need for structural and organizational improvements in the data and healthcare systems and provider workflows to achieve capacity‐building interventions for more robust changes in maternal and neonatal outcomes.

## AUTHOR CONTRIBUTIONS

SMH, SOS, ASM, and JGC were involved in the conception and design of the study. SAR, MKR, MTT, VSG, and PRG actively participated in the development of training materials, in the training, and also in data collection, under the supervision of SMH and ASM. SOS, SMH, and JGC were responsible for the analysis and interpretation of results. SOS wrote the first draft of the manuscript under the supervision of SMH and JGC. All authors read and agreed on the final version of the manuscript.

## FUNDING INFORMATION

No additional funds were available for this study except for the institutional support as recognized.

## CONFLICT OF INTEREST STATEMENT

The authors have no conflicts of interest.

## Data Availability

The raw data were generated at the Department of Obstetrics in the UNOESTE School of Medicine of Guarujá. Derived data supporting the findings of this study are available from the author, Dr. Samira M Haddad, on request.
